# Efficacy and safety of traditional Chinese patent medicine on carotid artery atherosclerosis in adults: A network meta-analysis protocol

**DOI:** 10.1097/MD.0000000000024406

**Published:** 2021-01-22

**Authors:** Huiqing Sun, Wei Qu, Guangjia Chen, Xiaonan Sun, Deqing Zhang, Shichuan Shao

**Affiliations:** a Tai’an City Central Hospital; b Tai’an Medical District of 960 Hospital of the Joint Logistics Support Force of the Chinese People's Liberation Army; c Tai’an City Cancer Prevention and Treatment Hospital, Tai’an, Shandong, China.

**Keywords:** atherosclerosis, protocol, systematic review, traditional Chinese patent medicine

## Abstract

**Background:**

Atherosclerosis (AS), the predominant pathological basis of ischemic cardiovascular and cerebrovascular diseases, remains a common and severe clinical problem. The experiments in vitro and in vivo indicate that Traditional Chinese patent medicine (TCPM) shows beneficial efficacy against AS through a variety of mechanisms. However, the existing therapeutic TCPM for the treatment of AS are diverse, and it is still significant to evaluate the pros and cons of a certain TCPM. Therefore, the study aims to compare the efficacy and outcomes of different anti-atherosclerotic TCPM in adults with the hope of providing references for clinical decision making.

**Methods:**

Cochrane Library, PubMed, Embase, Web of Science, China National Knowledge Infrastructure Database, Wanfang Database, Chinese BioMedical Literature Database, and China Science and Technology Journal Database will be searched. Randomized controlled trials (RCTs) of TCPM for aortic AS in adults will be included in this study if they meet the Population/Intervention/Comparison/Outcomes/Study Design (PICOS) criteria. Two reviewers will independently perform citations screening, data extraction and risk of bias assessment. STATA 15.0 and WinBUGS 1.4.3 will be employed to conduct statistical analyses under the Bayesian framework.

**Results:**

The efficacy and safety of various TCPM strategies on aortic AS in adults will be compared.

**Conclusion:**

The study will expand the range of options for anti-atherosclerotic therapeutic strategies and encourages further clinical research in traditional Chinese medicine.

**INPLASY registration number:**

INPLASY2020120036.

## 1 Introduction

Atherosclerosis (AS) is characterized by abundant fibrofatty lesions deposited in the arterial wall, and chronic inflammation occurs throughout.^[1,2]^ Lipids are deposited in the intima of elastic arteries and elastic muscle arteries, causing intimal fibrous thickening and deep tissues necrosis and disintegration. AS is a prevalent disease and plays a crucial role in cardiovascular and cerebrovascular diseases. Along with plaque enlargement, the arterial lumen undergoes a progressive narrowing process and downstream organs suffer from ischemia. Abrupt plaque rupture can lead to acute thrombotic events and thereby an interrupted blood flow cause hypoxic-ischemic cardio-cerebrovascular diseases.^[3,4]^ As the prominent killer of human health, heart disease, and stroke caused by AS are the 2 leading causes of death in the modern world.^[5,6]^ According to statistics, >17 million people died from heart disease in 2015, accounting for 31% of all deaths worldwide.^[5]^ Control of the initiation and progression of AS is therefore essential for cardiovascular and cerebrovascular diseases. Up to now, the pathogenesis of AS has not been fully elucidated, although there are many theories attempting to explain it. The main theories are lipid-derived theory, endothelial cell dysfunction, vascular smooth muscle micro-cloning, oxidative stress, immunometabolism, inflammation, gut microbiota theory, etc.^[7–12]^ At present, statins are essential for AS treatment and prophylaxis.^[13]^ Lipid modification therapy based on statins mainly functions by decreasing the level of low-density lipoprotein cholesterol (LDL-C). Although statins have achieved some success, blood lipids levels in some patients are still abnormal and often require intensive lipid-lowering therapy to attain the target LDL-C level. Besides, doubling the dose of statins or combining fibrates to strengthen lipid-lowering will increase the incidence of liver and kidney adverse reactions, or affect the cognitive function.^[14,15]^ On the other hand, the muscle aches due to statin intolerance in patients limit its clinical application.^[16]^ Atherosclerosis-triggered diseases are still of high morbidity and mortality. Therefore, the exploration of new prevention and treatment strategies for AS is still a long way off.

Traditional Chinese patent medicine (TCPM) has a definite curative effect and shows the advantages of reducing recurrence and side effects. Numerous studies have indicated that the combination treatment with TCPM and western medicine generates better efficacy than western medicine alone. Currently, TCPM have also actively participated in the treatment of AS. Commonly used TCPM for AS are Zhixiong Capsule, LongShengZhi Capsule, NaoXinTong Capsule, Danlou tablet, etc.^[17–20]^ Zhixiong Capsule (Leeches, Chuanxiong Rhizoma, *Salvia miltiorrhiza*, *Leonurus cardiaca*, Radix Pueraria, etc) can reduce LDL-C and total cholesterol (TC) levels and alleviate collagen deposition and mineralization by regulating IL-13, IL-4, MAPK1, MAPK14, P53, and JUN.^[18]^ LongShengZhi Capsule (Taxilluschinensis, Pheretima, Radix aucklandiae, *Acorus calamus*, *Carthamus tinctorius*, etc) exerts potential anti-thrombotic properties through AKT and ERK1/2 signaling pathway.^[19]^ NaoXinTong Capsule (Astragali Radix, Angelicae Sinensis, *Salvia miltiorrhiza*, Leeches, *Lumbricus rubellus*, etc) also shows anti-atherosclerotic effects by regulating lipid metabolism, anti-inflammatory, and anti-oxidative stress, and offers better tolerance and resistance than conventional western medicine.^[20]^ However, each independent randomized controlled trials (RCTs) has problems such as small sample size and single-center; at the same time, there is a lack of studies to compare the efficacy and safety of TCPM on AS.

Compared with 2-arm meta-analysis, the Network meta-analysis (NMA) can achieve a combination of direct and indirect comparison among multiple intervention factors. NMA is superior to a traditional meta-analysis in quantitative analysis of various interventions for the same disease and can screen an optimal intervention through quantitative ranking.^[21]^ Given this, this study adopts the method of NMA to evaluate the efficacy and safety of TCPM on carotid artery AS in adults to help make better clinical decisions.

## 2 Methods and analysis

### 2.1 Search strategy

The study will be conducted under the PRISMA-P guidelines to improve the transparency and repeatability of the research from a methodological level.^[22]^ By clarifying Population/ Intervention/ Comparison/Outcomes/Study Design (PICOS) issues, ensure that the literature can be retrieved scientifically. Additionally, the INPLASY registration number is INPLASY2020120036 (URL= https://inplasy.com/inplasy-2020-12-0036/).

Electronic research will be organized in PubMed, Cochrane Library, Embase, Web of Science, Chinese BioMedical Literature Database, China National Knowledge Infrastructure Database, China Science and Technology Journal Database, and Wanfang Database. The search time will be limited from inception of the database until November 2020. There is no languages and publication date restrictions. As a supplement to the study, the references in the included literature will be manually retrieved. We will use Endnote software for literature management. Search will be conducted combining thesaurus terms and free-text terms. The search terms are as follows:(traditional Chinese medicine or traditional medicine, Chinese or herbal medicine or herbs or Chinese medicine) and (Atheroscleroses or Atherogenesis or carotid Atherosclerosis or Carotid Atheroscleroses or Carotid Atherosclerotic Disease or Atherosclerotic Diseases, Carotid or Carotid Atherosclerotic Diseases or Atherosclerotic Disease, Carotid) and (randomized clinical trial or randomized or randomly).

### 2.2 Eligibility criteria

#### 2.2.1 Inclusion criteria

##### 2.2.1.1 Study design

The study included in the NMA is RCTs for adult AS.

##### 2.2.1.2 Participants

Patients are diagnosed with aortic atherosclerosis by vascular color Doppler ultrasonography. The diagnostic criteria for carotid artery plaque are defined as carotid artery intima-media thickness (IMT) ≥1.5 mm, which indicates plaque formation. Age ≥18, regardless of sex or race.

##### 2.2.1.3 Interventions

The control group received western medicine treatment, including lipid-lowering, antiplatelet aggregation, and other underlying diseases therapy. The experimental group receive the same western medicine treatment plus a TCPM for AS. Any mode of administration of TCPM will be included, such as oral and intravenous administration. There is no limit on the dosage and duration of intervention for TCPM.

##### 2.2.1.4 Outcomes

Primary outcome indicators: IMT, total carotid plaque area (TPA), Crouse plaque score, the incidence of adverse reactions.

Secondary outcome indicators: TC, triacylglycerol (TG), LDL-C, HDL-C levels.

#### 2.2.2 Exclusion criteria

The included patients have severe organic diseases and complications; in addition to TCPM, the experimental group is given any other treatment or drugs not used in the control group; literature information is incomplete or unclear; duplicate reports.

### 2.3 Data extraction

First, 2 researchers will independently screen titles and abstracts of each literature, exclude irrelevant literature, and decide which article to include. Second, data extraction will be undertaken from the original research with the consensuses by 2 independent investigators. The extracted information mainly consists of the following 4 aspects: identification of literature (title, journal, author, country and host institution of the study, publication year, study sponsorship); methodological characteristics (research design type, sample characteristics, specific details of the intervention for the control and experimental group, follow-up situation); outcome indicators and conclusions; key elements of risk assessment for bias. It is important to note that 2 investigators will cross-check the data throughout data extraction. If any disagreements appeared, a third reviewer would make a final decision.

### 2.4 Risk of bias assessment

Collaboration's risk of bias tool (Cochrane ROB) will assess the risk of bias.^[23]^ The instrument Cochrane ROB contains the following 6 items: selection bias; performance bias; detection bias; attrition bias; reporting bias; other potential bias. Risk of bias will be evaluated by 3 grades: low bias risk; uncertainty of bias risk; high bias risk. Two reviewers will independently conduct the risk of bias assessment. Any disagreement will be adjudicated by a third researcher.

### 2.5 Assessment of publication bias

Comparison-adjusted funnel plots will assess the publication bias according to the number of RCTs.

### 2.6 Data synthesis and statistical analysis

#### 2.6.1 Network meta-analyses

If the outcomes are measured as dichotomic variables, odds ratios (ORs) will be given with the 95% confidence interval (CI). If the outcomes are measured as continuous variables, standardized mean differences (SMD) will be given with the 95% CI. WinBUGS 1.4.3 software (MRC Biostatistics Unit, Cambridge, UK) will be used to fit the Bayesian hierarchical model. Three Markov chain Monte Carlo (MCMC) will run with 50,000 iterations and a burn-in period of 20,000. The network relationship graph will be plotted by STATA 15.0 software (Stata Corporation, College Station, TX). The thicker the line, the higher the number of RCTs compared between the 2 interventions. The bigger the dot, the larger the sample size of the RCTs. Surface under the cumulative ranking area (SUCRA) will be calculated. The larger the SUCRA values, the better the efficacy of interventions.

#### 2.6.2 Assessment of heterogeneity, transitivity, consistency

The heterogeneity test will conduct with I-square (*I*^2^) test. If *I*^2^ ≤ 50%, the heterogeneity has no statistical significance, a fixed effects model will be chosen. If *I*^2^ > 50%, we will look for the sources of heterogeneity. In the case where the heterogeneity cannot be excluded, a random-effects model will be applied. Describe the distribution of clinical and methodological variables by constructing box diagrams to evaluate transitivity.^[24]^ Meanwhile, we will use the node splitting approach to evaluate consistency if there is a closed loop.

#### 2.6.3 Subgroup analysis and sensitivity analysis

As for the subgroup analysis, we will analyze the sex, age, treatment period, and blood lipid to explore the influence of these factors on the total effect. Sensitivity analysis is to explore the impact on the total effect by excluding some studies with the poor methodologic quality or small sample size.

#### 2.6.4 Assessment of evidence quality

The Grading of Recommendations Assessment, Development and Evaluation (GRADE) will assess the evidence quality.^[25]^ The evidence quality will be classified in 4 grades: very low quality; low quality; moderate quality; high quality.

## 3 Discussion

AS progresses over time, with the ultimate event of a cardio-cerebrovascular disease. In recent decades, significant efforts have been made in AS prevention and treatment worldwide. As an alternative to western medicines, traditional Chinese medicines (TCM) have received increasing attention due to its prospective future.^[26]^ TCPM is made of natural Chinese herbal medicines and processed by special technology. Compared with TCM prescriptions’ complicated boiling process before to take, the application of TCPM is more convenient. At present, TCPM has been widely involved in the treatment of AS, and many studies have confirmed its efficacy. However, studies comparing the efficacy and safety of different TCPM on adult AS are currently lacking. This systematic review will summarize the evidence of TCPM for adult AS, and evaluate the efficacy and safety of a wide range of TCPM for aortic AS under the Bayesian framework. Considering that the NMA may be limited by the pooled data from RCTs, quality assessment and potential sources of heterogeneity will be considered with caution. Multicenter, large sample, high-quality RCTs are still in request, and we hope that high-quality NMA can make better choices for clinical treatment of adult AS.

## Author contributions

**Conceptualization:** Huiqing Sun, Shichuan Shao.

**Data curation:** Huiqing Sun, Wei Qu, Guangjia Chen.

**Formal analysis:** Wei Qu, Guangjia Chen.

**Investigation:** Wei Qu.

**Methodology:** Huiqing Sun, Shichuan Shao.

**Search strategy:** Huiqing Sun, Guangjia Chen, Xiaonan Sun.

**Software:** Xiaonan Sun, Huiqing Sun, Wei Qu.

**Statistical analysis:** Huiqing Sun, Guangjia Chen, Xiaonan Sun.

**Supervision:** Deqing Zhang.

**Visualization:** Xiaonan Sun.

**Writing – original draft:** Huiqing Sun.

**Writing – review & editing:** Shichuan Shao.
